# GnRH agonists induce endometrial epithelial cell apoptosis via GRP78 down-regulation

**DOI:** 10.1186/s12967-014-0306-y

**Published:** 2014-11-04

**Authors:** Huinan Weng, Fenghua Liu, Shuiwang Hu, Li Li, Yifeng Wang

**Affiliations:** Department of Pathophysiology, Key Laboratory of Proteomics of Guangdong Province, Southern Medical University, Guangzhou, China; GuangDong Women and Children Hospital, Guangzhou, China; ZhuJiang Hospital of Southern Medical University, Guangzhou, China

**Keywords:** Endometriosis, Eutopic endometrium, Gonadotropin-releasing hormone agonist, Proteomics, GRP78

## Abstract

**Background:**

Endometriosis is a benign chronic gynecological disease that affects women of reproductive age, characterized by the presence of functional endometrial tissues outside the uterine cavity. GnRH agonists exhibit anti-proliferative and apoptosis-enhancing activities and have long been used for the treatment of endometriosis. There is a critical need to identify the signaling modules involving GnRH agonist therapy for the treatment of endometriosis. In this study, we compared the proteomic profiles of endometriosis in patients before and after GnRH agonist therapy to identify proteins that might provide further information concerning the mechanisms underlying the functions of GnRH agonists.

**Methods:**

A total of 55 protein spots with different abundances were observed using Difference Gel Electrophoresis (DIGE), and 26 of these proteins were assigned clear identities through Matrix-Assisted Laser Desorption/Ionization Time-of-Flight Tandem Mass Spectroscopy (MALDI-TOF/TOF MS).

**Results:**

We validated four of these proteins through Western blotting and immunohistochemistry using human endometrial tissue. We also characterized the effect of Leuprolide acetate (LA) on the apoptosis of eutopic endometrial epithelial cells. LA treatment significantly promoted the apoptosis of eutopic endometrial epithelial cells and inhibited the expression of the anti-apoptotic factor GRP78. GRP78 knockdown enhanced LA-induced cell apoptosis, whereas, the overexpression of GRP78 in eutopic endometrial epithelial cells suppresses LA-induced apoptosis.

**Conclusion:**

These results suggest that GnRH agonists induce endometrial epithelial cell apoptosis via GRP78 down-regulation. This study might provide an important molecular framework for further evaluation of GnRH agonist therapy.

## Background

Endometriosis is a common benign chronic gynecological disease in which functioning endometrial glands and stroma are improperly present at sites outside the uterine cavity. This disease affects 5-15% of women of reproductive age, causes pain and dyspareunia, and is frequently associated with infertility [1]. Although endometriosis was first described in 1860, the etiology and pathogenesis of this disease remain uncertain. Within the past few years, important and promising advances have been made towards the development of new approaches to limit symptoms and improve fertility [2]. The current treatments for endometriosis primarily involve hormone suppressive therapies that down-regulate the hypothalamus–pituitary–ovarian pathway. These medical treatments effectively relieve symptoms of pain, nevertheless endometriosis exhibits a high recurrence rate and significant side effects, such as hot flashes and genital atrophy, are observed from the use of these medications [3].

Apoptosis, or programmed cell death, is a physiological process that plays a critical role in maintaining tissue homeostasis, killing unwanted cells without inducing immune responses or inflammatory reactions [4]. Apoptosis has been implicated in both normal development and disease [5]. Accumulating evidence suggests that apoptosis maintains cellular homeostasis during the menstrual cycle through the elimination of senescent cells from the functional layer of the uterine endometrium during the late secretory and menstrual phases of the cycle [6]. These findings suggest that apoptosis-inducing agents could be used as a potential therapeutic strategy for the treatment of endometriosis.

GnRH analogs (GnRHa) are the most widely used hormonal treatments for endometriosis [7]. Both GnRH and GnRH receptors have been isolated from the eutopic and ectopic endometrium, supporting a direct function for GnRHa in endometrial growth [8]. Other studies have also demonstrated that GnRHa induces apoptosis in both eutopic and ectopic endometrial cells from patients with endometriosis [7]. Similarly, it has been shown that GnRHa induces apoptosis and reduces cell proliferation in ovarian, endometrial and breast cancer cell lines [8,9]. Nevertheless, the effect of GnRHa treatment on the eutopic endometrium of patients with endometriosis and the activation of apoptosis through these events remain unknown.

Proteomic analysis is a powerful screening method for detecting unexpected changes in protein expression that might be missed using conventional biochemical techniques. Aside from accelerating diagnosis, the use of proteomics has enhanced the current understanding of the physiopathological progression of endometriosis through the identification of proteins involved during different stages [10]. In the present study, we compared the proteomic profiles of endometriosis in patients before and after GnRH agonist therapy to identify proteins involved in the mechanisms of GnRH agonist action. We detected 55 protein spots with different abundances via Difference Gel Electrophoresis (DIGE), and 26 of these proteins were assigned clear identities using MALDI-TOF/TOF MS. Subsequently, we validated four of these proteins through Western blotting and immunohistochemistry on human endometrial tissue and investigated the effect of Leuprolide acetate on the apoptosis of eutopic endometrial epithelial cells. Leuprolide acetate significantly promoted the apoptosis of eutopic endometrial epithelial cells and inhibited the expression of anti-apoptotic factor GRP78. These results suggest that Leuprolide acetate induces endometrial epithelial cell apoptosis throught the downregulation of GRP78.

## Materials and methods

### Patients

A total of 14 patients with endometrioma and pelvic endometriosis were recruited from 2012 to 2013. The clinical characteristics of the patients are shown in Table 1. Endometriosis was determined laparoscopically. The severity of endometriosis in all cases was classified according to the Revised American Fertility Society rating (r-AFS; 1985). Four stage III and ten stage IV were included in the present study. This study was approved through the Ethics and Research Committee of the Guangdong Women and Children’s Hospital, Guangzhou, China. All subjects provided written informed consent.

**Table 1:** **Clinical characteristics of the patients enrolled in this study**

**Age (years)**	**Day of cycle before GnRHa therapy**	**Stage of endometriosis**
(a) Tissue for DIGE assay (n = 4)		
33	15	IV
27	16	IV
27	18	IV
37	27	III
(b) Tissue for WB assay (n = 10)		
33	10	IV
25	14	IV
32	17	IV
29	13	III
27	15	IV
26	13	III
29	15	IV
23	11	IV
39	9	IV
36	12	III

Stages of endometriosis according to the Revised American Fertility Society score (r-AFS). WB: Western blot analysis.

### Endometrial tissues

Women with abnormal periods or exposure to steroids within 3 months before surgery were excluded. The eutopic endometrium samples were collected through curettage during laparoscopic surgery (control group, n = 14). After surgery, GnRHa was administered as supplemental therapy to prevent recurrence. Each patient received an intramuscular injection with 3.75 mg of Diphereline (Triptorelin Acetate for injection, Ipsen, Pharma Biotech, France) on the first day of the first menstruation after surgery. On the 28th day after the first injection, the same patient underwent hysteroscopy, and a eutopic endometrium sample was also collected.

### Sample preparation

The samples were frozen in liquid nitrogen and preserved at −80°C. The frozen endometrial tissue biopsy samples were weighed (100 mg/mL lysis buffer) and immediately thawed in phosphate-buffered saline solution on ice. The tissues were washed five times with phosphate-buffered saline solution to remove adhered hemoglobin. The dialyzed samples were resuspended in Lysis Buffer (30 mM Tris–HCl, 7 M urea, 2 M thiourea, and 4% CHAPS, pH 8.5) and incubated on ice for 30 min. The suspensions were sonicated on ice to prevent sample heating, and a total of five 10-second bursts with 30-second pauses were used. The lysates were subsequently centrifuged at 12,000 g for 30 minutes. The proteins were precipitated from the suspension using the 2D Clean-up Kit (GE Healthcare) according to the manufacturer’s instructions and resuspended in Lysis Buffer. The protein concentration was determined using the 2D Quant Kit (GE Healthcare) according to the manufacturer’s instructions. The proteins were aliquoted followed by freezing or freeze-drying. All reagents were obtained from the Sigma Chemical Company unless otherwise noted.

### Differential in-gel electrophoresis (DIGE)

For DIGE, 50 μg of protein were minimally labeled with Cy Dyes at the ratio of 50 μg of protein: 400 pmol Cy3 or Cy5 protein-labeling dye (GE Healthcare) according to the manufacturer’s instructions. Cy3 and Cy5 were used to label the samples, and Cy2 was used for the internal standard (a pool of all samples). Each labeled sample was mixed with rehydration buffer (7 M urea, 2 M thiourea, 4% CHAPS, 2% dithiothreitol, and 2% Pharmalyte; GE Healthcare) and applied to a 24-cm immobilized pH gradient gel strip (immobilized pH gradient (IPG) strip pH 3 to 10 NL) for separation in the first dimension. First-dimension isoelectric focusing was performed at 20°C using the IPGphor III System (GE Healthcare) and the following protocol: 30 V for 12 hours; 1,000 V gradient for 2 hours; 8,000 V gradient for 2 hours; and 8,000 V for 60,00 volt-hours, followed by a 500 V hold. After isoelectric focusing, the strips were equilibrated through agitation for 15 minutes in 50 mM Tris–HCl, pH 8.8, 6 M urea, 30% (v/v) glycerol, 2% (w/v) sodium dodecyl sulfate (SDS) and 650 mM DTT, followed by agitation for 15 min in 50 mM Tris–HCl, pH 8.8, 6 M urea, 30% (v/v) glycerol, 2% (w/v) SDS and 1.27 M iodoacetamide. The strips were subsequently loaded onto a 24 × 24 cm 12% polyacrylamide gel using low fluorescence glass plates and subjected to an electric field in DALT Six (GE Healthcare; 15°C at a constant 3 W per gel for 12 hours in a running buffer containing 25 mM Tris, 192 mM glycine, and 0.1% (w/v) SDS). Subsequently, the gels were scanned on a Typhoon 9400 imager (GE Healthcare) and analyzed using DeCyder 2D Software V6.5 (GE Healthcare). The differentially expressed protein spots observed between groups (filtering conditions: at least 50% change of ratios between groups and t test *P* <0.05) were considered for further examination. The samples for the spot picking gel were prepared without Cy dye labeling, and a 600 μg of pooled protein sample was run on a preparative gel, followed by staining with colloidal Coomassie Blue G-250. The matched spots were automatically selected from the preparative gel using an Ettan Spot Picker (GE Healthcare).

### Protein identification

The selected spots were destained with 50% acetonitrile (ACN)/100 mM NH_4_HCO_3_ for 10 min, dehydrated with 100% ACN for 10 min, and dried using a centrifugal concentrator (TOMY SEIKO, Tokyo). Next, the gel slices were incubated for 30 min at 4°C with 2 μL of 25 ng/mL trypsin (Promega) diluted in 50 mM NH_4_HCO_3_. Subsequently, 30 μL of 50 mM NH_4_HCO_3_ was added, and the spots were further incubated overnight at 37°C. We used two solutions to extract the resulting peptide mixtures from the gel slices. First, 100 μL of 60% (v/v) ACN in 0.1% aqueous trifluoroacetic acid (TFA) was added, and the mixture was sonicated for 15 min. Next, we collected the solution and added 50 μL of 100% ACN for the final extraction. The digested peptides were dried using a vacuum pump, dissolved in 2 μL 50% acetonitrile/0.1% TFA, and 0.5 μL of the peptide sample was loaded onto the target disk and air-dried. Subsequently, 0.5 μL of matrix solution (CHCA saturated in 50% acetonitrile/0.1% TFA) was added to the dried samples and dried. The samples on the MALDI target plates were analyzed using an ABI 4800 Proteomics Analyzer MALDI-TOF/TOF mass spectrometer (Applied Biosystems). A total of 800 shots were accumulated for MS analyses. The MS/MS analyses were performed using air, at 2-KV collision energy. The MASCOT search engine (version 2.1, Matrix Science) was used to search the tandem mass spectra. GPS Explorer™ software version 3.6.2 (Applied Biosystems) was used to create and search files, and the MASCOT search engine was used for peptide and protein identification against Swiss-Prot non-redundant sequence databases selected for human taxonomy.

### Western blot

The differentially expressed proteins, GRP78 (glucose-regulated protein 78 kDa), PPA1 (pyrophosphatase (inorganic) 1), EFHA2 (EF-hand domain family, member A2), and TGM2 (Protein-glutamine gamma-glutamyltransferase 2), were further validated through Western Blot analysis, using GAPDH (diluted 1:1000, Santa Cruz, USA) as the loading control. Equal amounts of protein (50 μg), extracted from 10 samples as previously described, were loaded and subjected to 12.5% SDS-PAGE. The proteins were subsequently transferred to PVDF membranes. The membranes were blocked for 1 hour at room temperature in TBST containing 5% skim milk. Rabbit polyclonal antibodies against GRP78, PPA1, EFHA2 and TGM2 (diluted 1:1000, Santa Cruz, USA) were used as primary antibodies. After washing 3 times with TBST for 5 min each time, the membranes were incubated with each primary antibody at room temperature for 2 hours. The membranes were washed again, followed by incubation with a secondary peroxidase-conjugated antibody (diluted 1:10000; Abcam, USA) for 1 hour at room temperature. After washing with TBST, the reaction was detected through chemiluminescence using ECL reagents (Forevergen Biosciences, China). The membranes were exposed and scanned using a Carestream Image Station 4000R (Carestream Health, USA). A semi-quantitative analysis based on OD was performed using Quantity One software (Bio-Rad). Paired samples *t*-test was used to determine the mean differences between the two groups, and a *P* <0.05 was considered significant at a 95% confidence level.

### Immunohistochemistry

Ectopic and eutopic endometrial tissue sections were fixed in 4% buffered paraformaldehyde in saline for 4 h at 4°C and processed using standard procedures. Paraffin sections (5 μm) were used for the immunohistochemical localization of GRP78, PPA1, EFHA2, and TGM2 proteins. The tissue sections were incubated overnight at 4°C with appropriate concentrations of the specific antibodies according to the manufacturer’s recommendations. Subsequently, the tissue sections were further incubated with the secondary antibody (biotinylated IgG) for 45 min at room temperature. Serum or IgG from the respective species with reference to the primary antibody at the respective dilution was used for the negative control. Visualization was achieved using the Vectastain ABC Peroxidase Elite Kit (Vector Laboratories, Burlingame, CA, USA) and freshly prepared 3,3′-diaminobenzidine (DAB) tetrahydrochloride (Sigma Chemical Company, St Louis, MO, USA) as a substrate. The staining intensity was quantified for each protein using Image-pro Plus according to the manufacturer’s instructions (Media Cybernetics, Inc., Bethesda, MD).

### Apoptotic assay

For the in vivo apoptosis assay, eutopic endometrial tissue sections were fixed in 4% paraformaldehyde for 24 h at 4°C, washed in phosphate-buffered saline (PBS), and embedded in paraffin blocks. The tissue sections were incubated with proteinase K (20 μg/mL, without DNAase), washed three times with HBSS, and subsequently labeled using the TdT-FragEL™ DNA fragmentation detection kit (Calbiochem), according to the manufacturer’s instructions. As a negative control, Tris-buffered saline (TBS) was substituted for the TdT enzyme. Each section was analyzed in at least 3 continuous fields of vision at high magnification, and the number of apoptotic cells per 1,000 cells in each field was recorded. The TUNEL-positive cells were counted from more than 10 areas and averaged in each anatomic region using high-power-field (HPF, ×200) light microscopy.

For the in vitro apoptosis assay, the percentage of apoptotic cells was also assessed through TUNEL in endometrial cultures under basal conditions and after exposure to Leuprolide Acetate (LA) (25, 50, and 100 ng/mL).

### Primary eutopic endometrial epithelial cell culture

The tissue was immediately placed in culture medium and processed within 60 min of collection. The epithelial cells were enzymatically separated and isolated through successive centrifugation. The cells were maintained in Dulbecco’s modified Eagle’s medium (DMEM)/F12 medium supplemented with 5% fetal bovine serum, 100 U/mL penicillin G and 100 g/mL streptomycin (Life Technologies, Grand Island, NY, USA) at 37°C in a humidified atmosphere of 5% CO_2_ and 95% air.

### Transfection of GRP78 in eutopic endometrial epithelial cells

After the cells reached 70-80% confluence, the medium was removed and the cells were placed in serum-free OPTI-MEM medium containing siRNA according to the manufacturer’s instructions. Two small interfering (si) RNAs targeting human GRP78 (siGRP78-1 and siGRP78-2) were designed using the following primer sequences: 5′-CGACUCGAAUUCCAAAGAUdTdT-3′ and 5′-GAUAAUCAACCAACUGUUAdTdT-3′, respectively. The GRP78 protein expression in siRNA-transfected cells was analyzed through Western blotting. To determine whether GRP78 inhibits Leuprolide acetate-induced endometrial epithelial cell apoptosis, eutopic endometrial epithelial cells were seeded onto 6-well plates and transfected the following day with pCMV-3X Flag-GRP78 or the pCMV-3X Flag vector alone using Lipofectamine 2000 (Invitrogen). The effect of GRP78 overexpression on Leuprolide acetate-induced endometrial epithelial cell apoptosis was assessed using a TUNEL assay.

### Statistical analysis

The statistics features in DeCyder were used to evaluate the results of the 2-DE gels. For all protein spots, the GnRHa^(+)^/GnRHa^(−)^ groups were compared, and the differences were calculated as changes in the volume ratios using Student’s *t*-test as selection criteria. The protein spots with differential expression between the groups (filtering conditions: at least 50% change of ratios between groups and *t*-test *P* <0.05) were extracted. For western blot (WB) analysis, paired samples *t*-test was used to determine the mean differences between the two groups, and a *P* <0.05 was used to assess the significance of these differences using SPSS13.0 software.

## Results

### GnRHa induces eutopic endometrial epithelial cell apoptosis in patients with endometriosis

**Figure 1:**
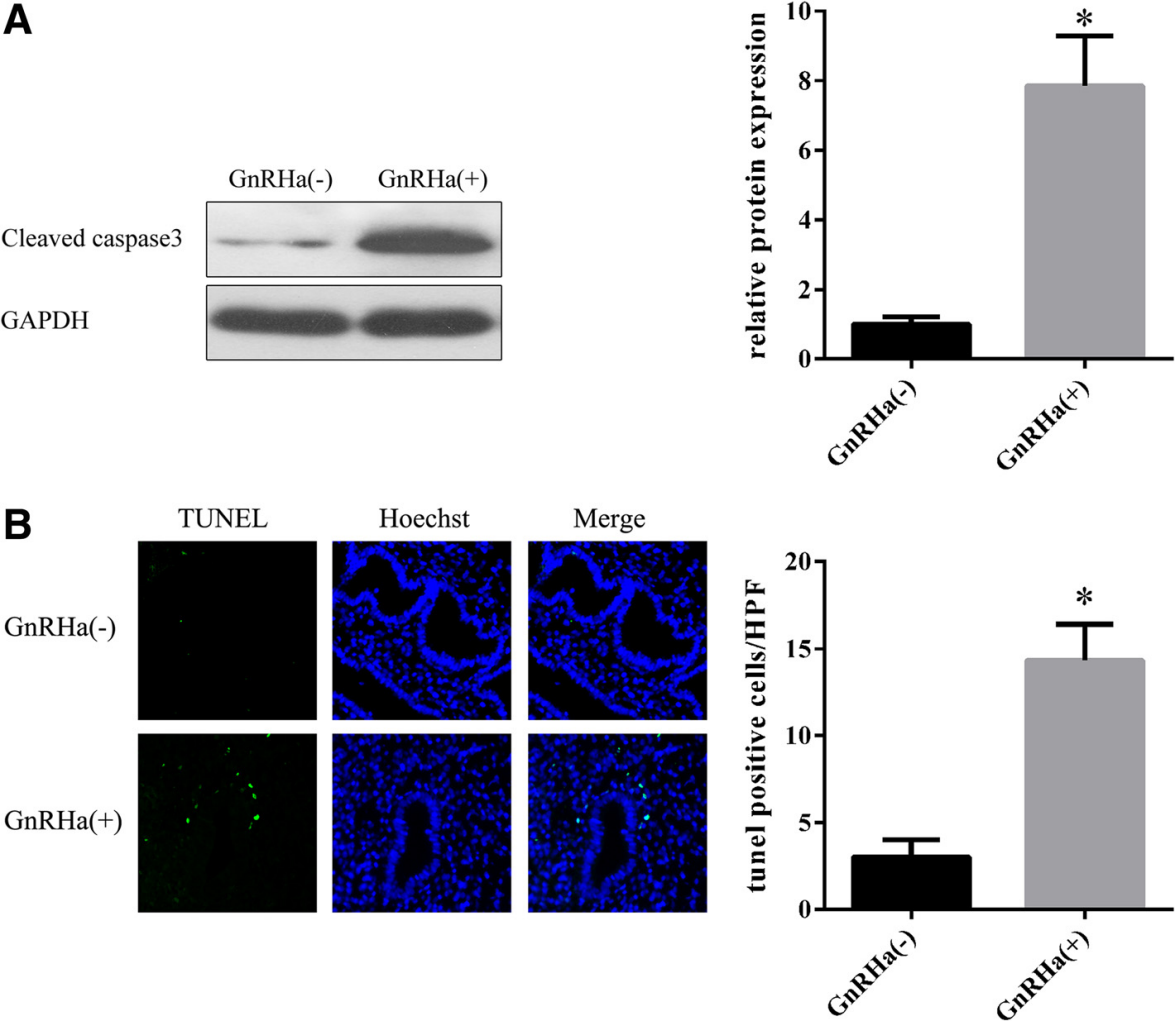
**Triptorelin acetate induces eutopic endometrial epithelial cell apoptosis in patients with endometriosis. A**. Western blot analysis of cleaved Caspase 3 in eutopic endometrial epithelial cells from endometriosis patients after Triptorelin acetate treatment. **B**. TUNEL assay to detect apoptosis in eutopic endometrial epithelial cells from endometriosis patients after Triptorelin acetate treatment. *P <0.05.

To characterize the signaling pathway in endometriosis pathogenesis and the mechanisms underlying GnRHa therapy, we determined the apoptosis levels in endometriosis before and after GnRHa treatment (by intramuscular injection with Triptorelin Acetate) (Figure 1). Western blot analysis showed that compared with the non-treated control group, cleaved Caspase 3 expression in endometriosis patients after Triptorelin acetate treatment was higher than that in GnRHa-control group. The results of the TUNEL assay showed that the apoptotic rate in the GnRHa-treated group was significantly higher compared with the GnRHa non-treated group (*P* <0.05).

### Proteomics assay

**Figure 2:**
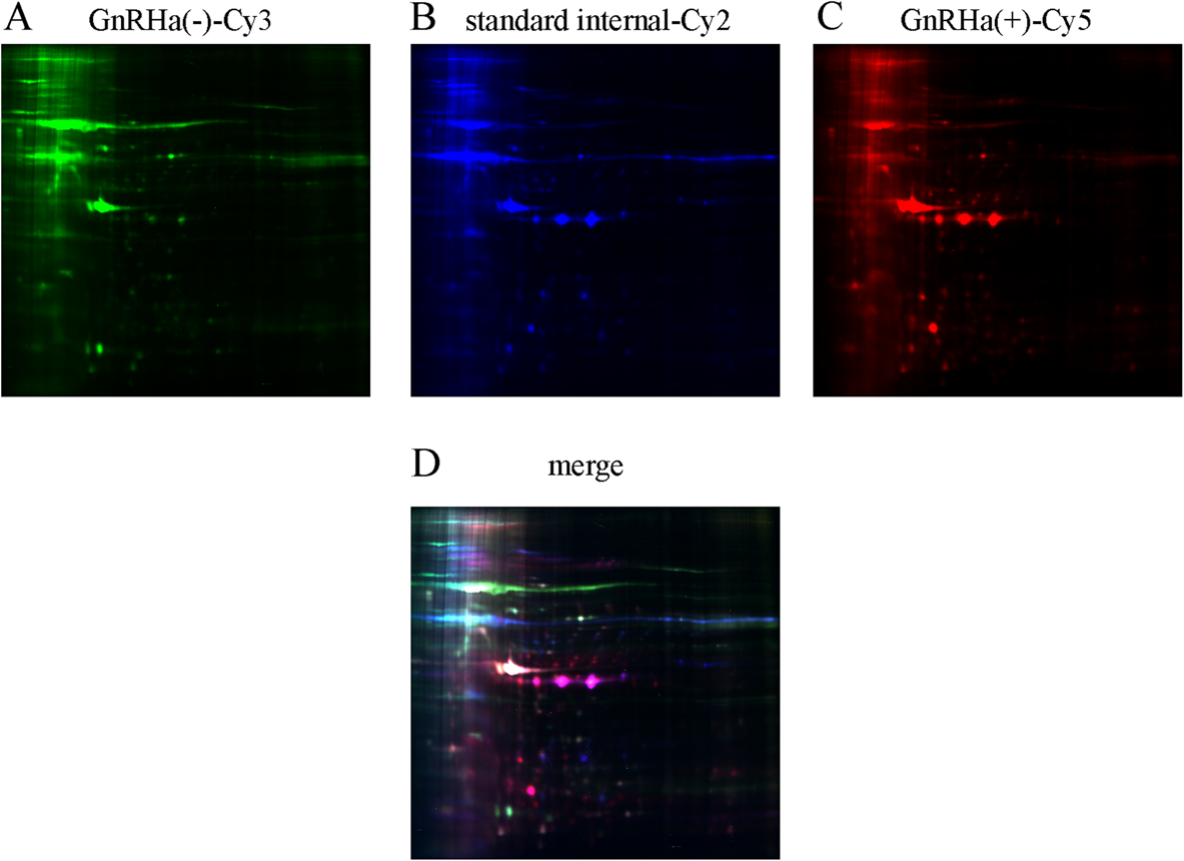
**2D**-**DIGE of eutopic endometrial proteins.** Each individual sample (before and after GnRHa treatment) and internal reference sample was labeled with Cy5, Cy3 and Cy2, respectively, mixed, and separated on a 2D-PAGE gel. The gels were scanned, and Cy5 **(A)**, Cy3 **(B)** and Cy2 **(C)** images were obtained from each gel. An overlay of three dye-scanned images was also obtained **(D)**.

To analysis differentially expressed proteins in the eutopic endometrium of endometriosis patients before and after GnRHa treatment, we used 2-DE separation. Representative results are shown in Figure 2. A total of 55 protein spots with different abundances were identified through DIGE, of which 26 altered proteins were identified using MALDI-TOF/TOF MS. A total of 12 proteins were up-regulated and 14 proteins were down-regulated (Table 2). These proteins included 6 secreted proteins, 4 cytoskeletal proteins, 4 molecular chaperones, 4 proteins with catalytic activity, 2 proteins involved in redox reactions, and 2 cell membrane proteins to name a few. Actin, keratin, the Tubulin alpha-1B chain and the Tropomyosin alpha-4 chain were among the cytoskeletal proteins identified. Endoplasmin, a 78-kDa glucose-regulated protein, a 60-kDa heat shock protein and T-complex protein 1 subunit epsilon were among the molecular chaperones identified. The proteins involved in redox reactions included Peroxiredoxin-4 and Thioredoxin-related transmembrane protein 1. After the first administration of GnRHa therapy, Peroxiredoxin-4 protein was highly expressed. Moreover, the cytoskeletal proteins were up-regulated, and the molecular chaperones were down-regulated.

**Table 2:** **Eutopic endometrial proteins showing significant differences in expression before and after GnRH agonist therapy**

**Gene name**	**Uniprot access no.**	**MW** **(kDa)**	**PI**	**Ratio** **(treat:** **control)**
HSPA5	P11021(GRP78)	72.4	5.07	−4.17
HSP90B1	P14625	92.7	4.76	−3.74
CCT5	P48643	60.1	5.45	−2.67
PDIA6	Q15084	48.5	4.95	−2.39
LMNA	P02545	74.4	6.57	−2.32
SASH1	O94885	137.7	5.78	−2.23
ALB	P02768	71.3	5.92	−2.2
PPA1	Q15181	33.1	5.54	−2.11
HSPD1	P10809	61.2	5.7	−1.94
TMX1	Q9H3N1	32.2	4.92	−1.85
FGB	P02675	56.6	8.54	−1.76
APOA1	P02647	30.8	5.56	−1.72
PDIA3	P30101	57.1	5.98	−1.7
DCN	P07585	40.1	8.75	−1.6
PHB2	Q99623	33.3	9.83	1.55
KRT1	P04264	66.2	8.15	1.6
TPM4	P67936	28.6	4.67	1.68
FGG	P02679	52.1	5.37	1.77
PRDX4	Q13162	30.7	5.86	1.78
TUBA1B	P68363	50.8	4.94	1.92
FTL	P02792	20.1	5.51	2.03
FGA	P02671	95.7	5.7	2.21
EFHA2	Q86XE3	61	8.35	3.95
TGM2	P21980	78.4	5.11	4.69
SIGLEC16	A6NMB1	53.8	9.28	6.05
ACTG1	P63261	42.1	5.31	6.68

### Validation of selected candidates through immunohistochemistry and western blotting

**Figure 3:**
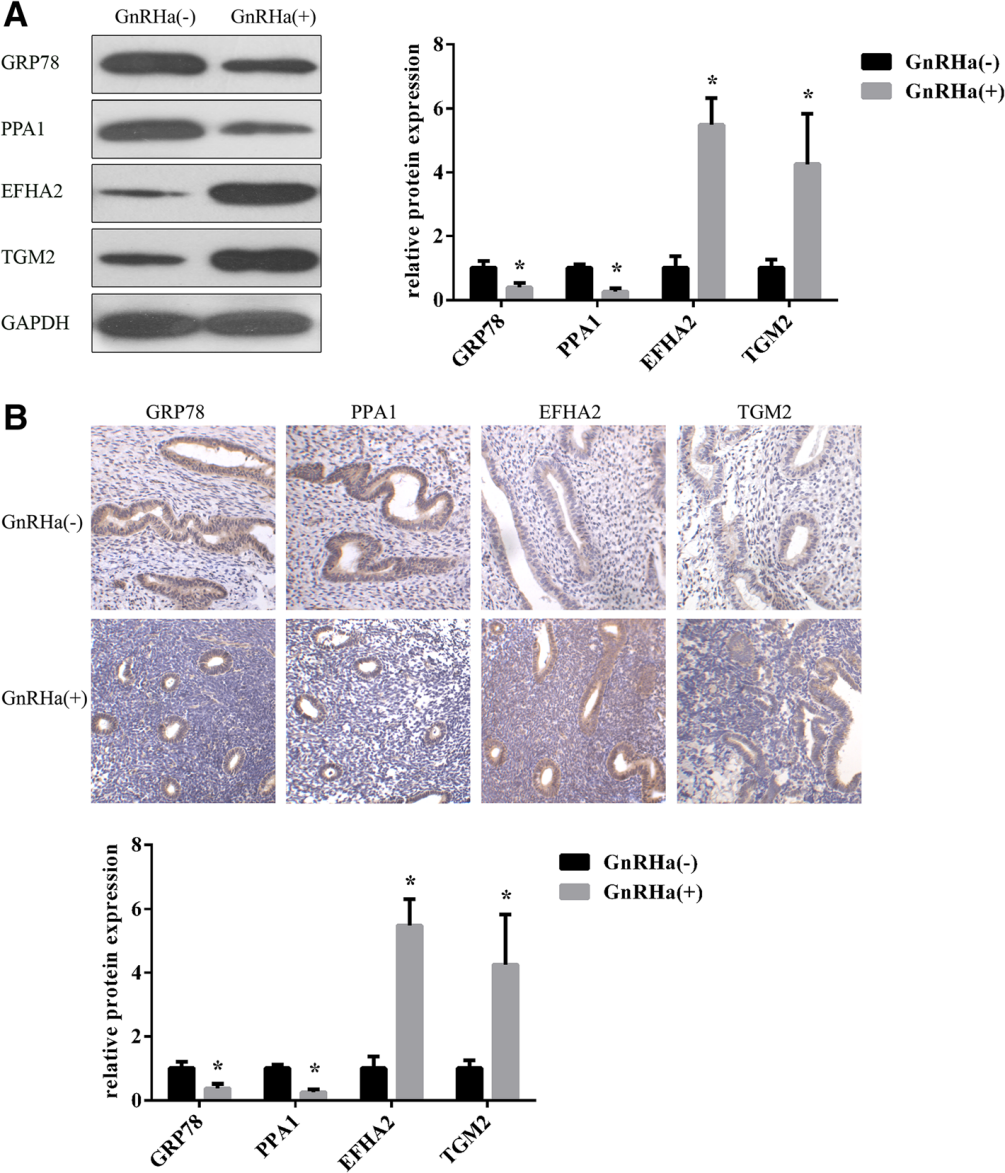
**GRP78**, **PPA1**, **EFHA2**, **and TGM2 proteins expression in tissues before and after GnRHa treatment. A**: Western blot analysis of 4 identified proteins (GRP78, PPA1, EFHA2, and TGM2) in eutopic endometrial epithelial tissues before and after GnRHa treatment (n = 10). The statistical analysis of the protein expression levels in eutopic endometrial epithelial tissues is shown on the right. **B**: Immunohistochemical detection of GRP78, PPA1, EFHA2, and TGM2 proteins in eutopic endometrial epithelial tissues before and after GnRHa treatment (n = 10). *P <0.05.

To validate the results of the DIGE analysis, four proteins, selected on the basis of interesting biological functions and expression changes, were examined in western blotting experiments using commercially available specific antibodies on protein extracts from endometriosis patients before and after GnRHa treatment. As shown in Figure 3A, the levels of GRP78 and PPA1 expression were higher in the GnRHa- (untreated) group compared with the GnRHa + (GnRHa-treated) group (*P* <0.05), while EFHA2 and TGM2 expression significantly increased in endometriosis patients after GnRHa treatment (*P* <0.05).

To further confirm the differential expression observed in 2D-DIGE screening, we also performed immunohistochemistry (IH) staining using paraffin sections.

As shown in Figure 3B, the results of IH were consistent with the expression changes observed through 2D-DIGE and western blot analysis.

### Effects of GnRHa on epithelial cell apoptosis

**Figure 4:**
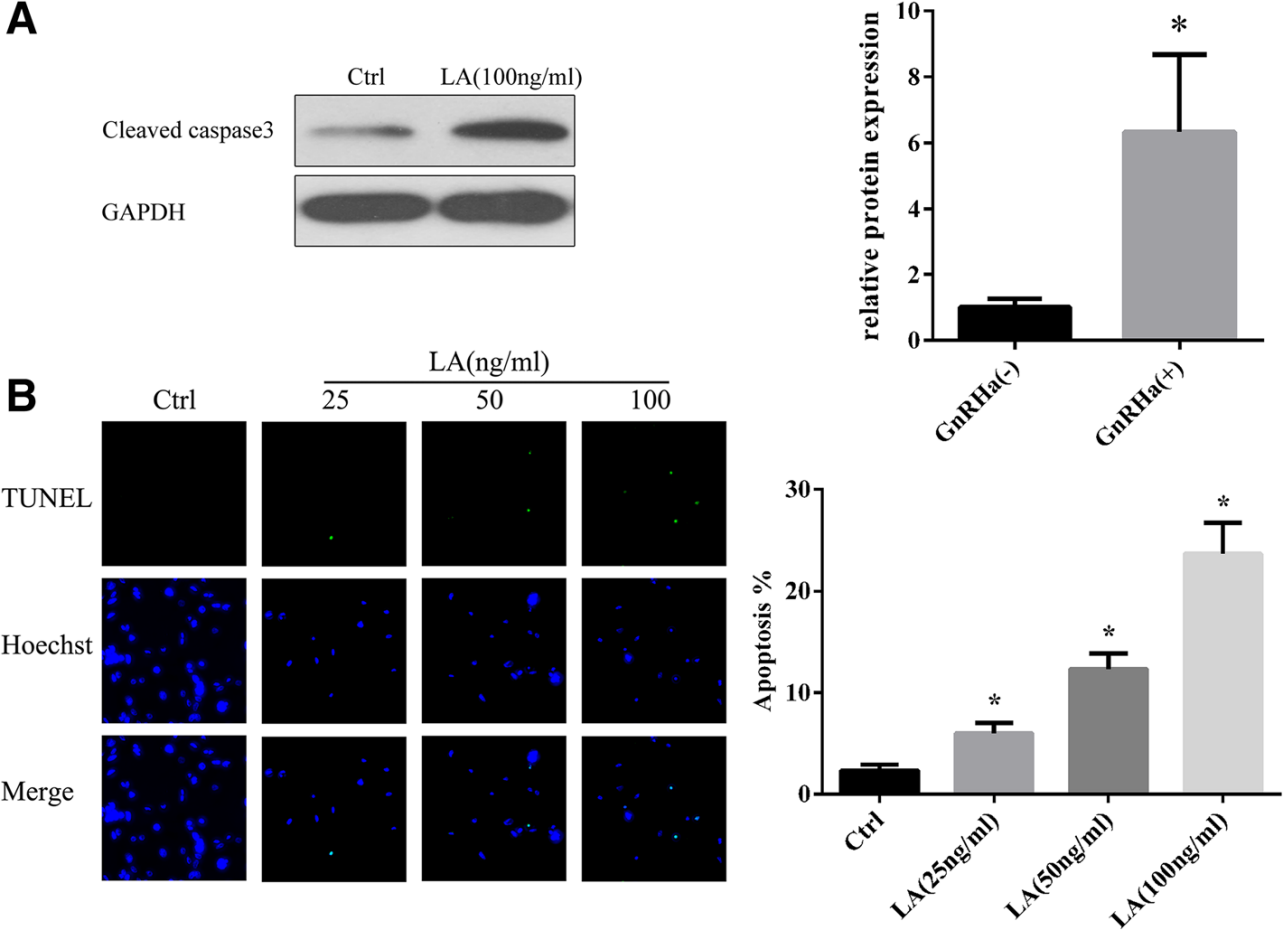
**Leuprolide acetate induces eutopic endometrial epithelial cell apoptosis. A**: Leuprolide acetate (100 ng/mL) up-regulated the expression of cleaved Caspase 3. **B**: Apoptosis detected through TUNEL staining. Compared with the normal group (untreated), the rate of apoptosis in eutopic endometrial epithelial cells after treatment with 25, 50 and 100 ng/mL Leuprolide acetate was statistically significant. *P <0.05.

For the in vitro functional analyses, Leuprolide Acetate was used for GnRHa treatment because of its similar common mechanism of action and pharmacological effects with Triptorelin and is widely use in similar studies [11,12]. The effects of different LA concentrations on epithelial cell apoptosis are shown in Figures 4A and 4B. Western blot analysis showed that cleaved Caspase 3 expression in epithelial cells after LA treatment was higher than that in the control group. (Figure 4A). We also observed that 25 ng/mL of LA enhanced epithelial cell apoptosis from 2.33 ± 0.33 to 6.00 ± 0.58% (expressed as a percentage of apoptotic cells, *P* <0.05) (Figure 4B), and the concentration-dependent stimulation of cell apoptosis (6.00 ± 0.58, 12.33 ± 0.88, and 23.67 ± 1.76%) using 25, 50, and 100 ng/mL of LA, respectively, was also observed.

### GRP78 knockdown enhanced LA-induced cell apoptosis

**Figure 5:**
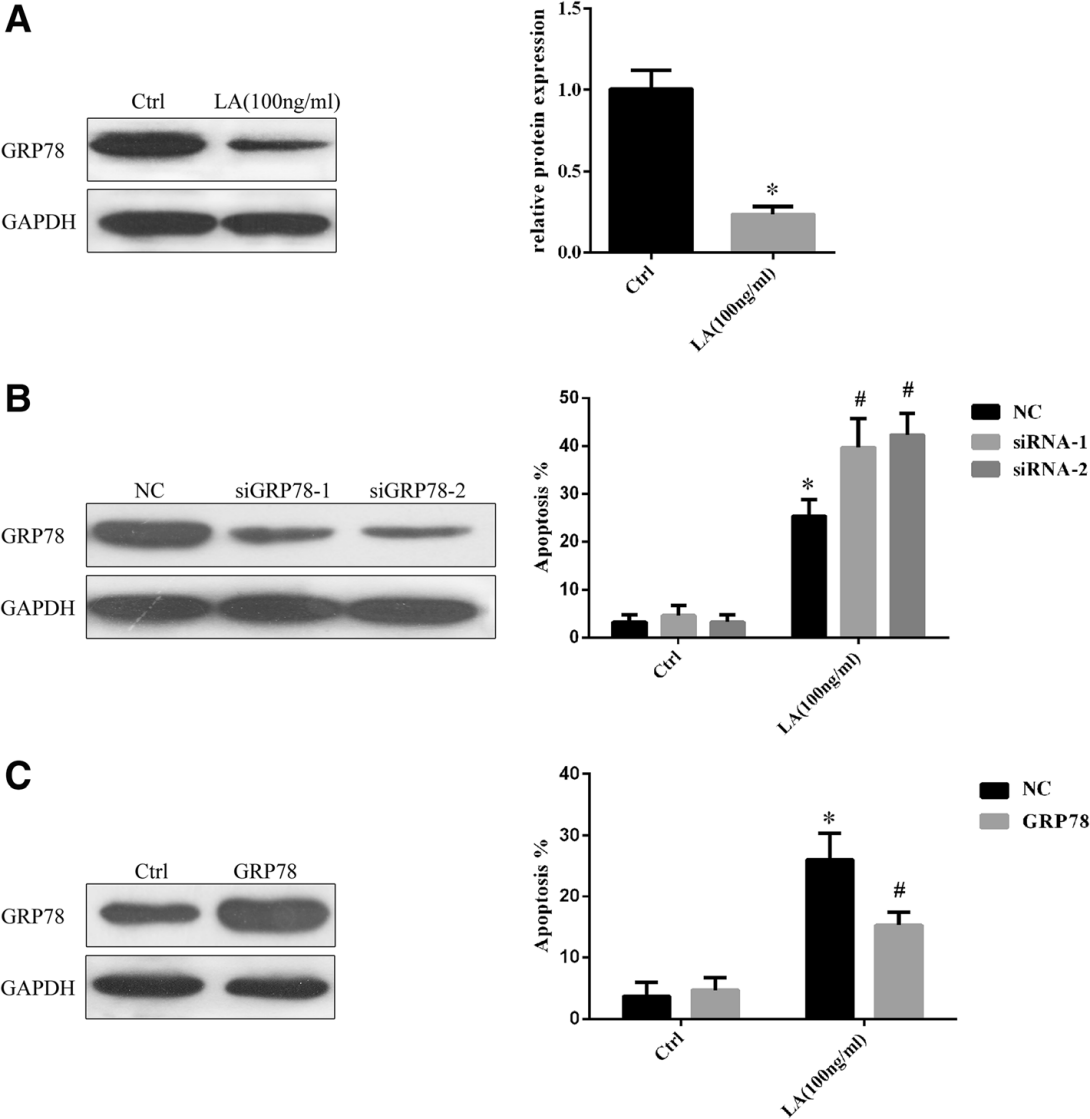
**Influence of GRP78 knockdown and overexpression on eutopic endometrial epithelial cells apoptosis. A**: GRP78 expression in separated eutopic endometrial epithelial cells before and after GnRHa treatment was detected through Western blotting. The statistical analysis of the two groups showed obvious differences. *P <0.05 **B**: The GRP78 knockdown cells exhibited lower GRP78 expression than the control cells. (Left) The knockdown of GRP78 did not affect normal endometrial cell apoptosis, while Leuprolide acetate treatment increased the percentage of apoptosis in the GRP78 knockdown cells compared with the control cells (Right). Ctrl, cells without LA treatment; NC, cells transfected with scrambled control; siRNA-1, cells transfected with siRNA-1 that targeting GRP78; siRNA-2, cells transfected with siRNA-2 that targeting GRP78. *P <0.05 vs NC within the Ctrl goup, #*P* <0.05 vs NC within the LA goup. **C**: Comparison of the GRP78 protein levels in GRP78 overexpression and control groups. (Left); GRP78 overexpression did not affect normal endometrial cell apoptosis, while GRP78 overexpression protected the cells from Leuprolide acetate-induced apoptosis (Right). Ctrl, cells without LA treatment; NC, cells transfected with pCMV-3X Flag; GRP78, cells transfected with pCMV-3X Flag-GRP78. *P <0.05 vs NC within the Ctrl goup, #*P* <0.05 vs NC within the LA goup.

GRP78 expression in isolated eutopic endometrial epithelial cells before and after GnRHa treatment was detected through Western blotting. As shown in Figure 5A, the GRP78 expression after LA treatment was significantly decreased in eutopic endometrial epithelial cells compared with the control group. Figure 5B shows the expression of GRP78 in scrambled control and GRP78 knockdown eutopic endometrial epithelial cells. GRP78 knockdown epithelial cells exhibited lower GRP78 expression than the scrambled control epithelial cells. We further evaluated cell apoptosis after LA treatment in GRP78 knockdown and scrambled control epithelial cells. The percentage of apoptotic GRP78 knockdown cells (39.67 ± 6.11 and 42.33 ± 4.51 in siRNA-1 siRNA-2 cells, respectively) was much higher in the scrambled control cells (25.33 ± 3.51, *P* <0.05) at 24 h after LA treatment (Figure 5B, right). These results suggest that GRP78 is a target protein for LA-induced cell apoptosis in endometriosis.

### The overexpression of GRP78 in eutopic endometrial epithelial cells suppresses LA-induced apoptosis

To determine whether GRP78 protects cells from LA-induced apoptosis, we transfected pCMV-3 × Flag-GRP78 into eutopic endometrial epithelial cells to obtain the overexpression of GRP78. Immunoblot analysis followed by normalization against GAPDH revealed a 3-fold increase in the level of GRP78 expression in transfected cells compared with non-transfected cells (Figure 5C, left). Although the overexpression of GRP78 did not affect normal endometrial cell apoptosis, the percentage of apoptotic GRP78-overexpressing cells (15.33 ± 2.08) was much lower than that observed in the scrambled control cells (26.00 ± 4.36).

## Discussion

The eutopic endometrium of patients with endometriosis is fundamentally different that of women without this disease. The apoptosis indices in the eutopic endometrium of endometriosis patients were lower than those in women without endometriosis [13]. The increased cell viability observed in endometriosis reflects a reduction in cell death through apoptosis and an increase in cell proliferation, suggesting that this condition might facilitate the invasive feature of the endometrium [14]. GnRH analogs enhanced EEC apoptosis, accompanied by an increase in the expression of the pro-apoptotic proteins Bax and FasL and a decrease in the expression of the anti-apoptotic protein Bcl-2 [12]. These observed differences suggest that the eutopic endometrium of women with endometriosis is inherently aberrant and plays an important role in the pathogenesis of this disease [13,15]. GnRHa therapy has been widely used for the treatment of estrogen-dependent disorders, such as endometriosis. These compounds markedly suppress the gonadotropin output from the pituitary gland, causing hypoestrogenism and a reduction in the size of endometriotic lesions. However, the local anti-proliferative effects of GnRHa might act not only through the suppression of gonadal steroids, but also through a direct effect on cell apoptosis [16]. There is strong evidence showing that GnRHa directly induces the apoptosis of endometrial and several types of cancer cells [7,12,17].

In the present study, to validate the apoptosis-promoting effect of GnRHa therapy on eutopic endometrial epithelial cells, we determined the level of apoptosis in endometriosis before and after GnRHa treatment. The results of Western blotting and TUNEL assays showed that GnRHa induces eutopic endometrial epithelial cell apoptosis in patients with endometriosis. This finding is consistent with Imai et al. [18], who reported that a GnRH agonist triggered apoptosis in a single suspension of stromal and glandular epithelial endometrial cells from patients with endometriosis and a recent study reporting that GnRH-a stimulates apoptosis in endometrial cells from patients with symptomatic myomas and this effect could, at least in part, account for the therapeutic action of GnRHa [19]. In addition, we also used epithelial human endometrial cell cultures as a model to evaluate cell apoptosis in response to the addition of GnRHa. Exposure to LA significantly increased the level of apoptosis in cell cultures from patients and controls in a concentration-dependent manner. Taken together, these results strongly suggest that GnRHa might effectively promote the apoptosis of endometrial cells in vitro and in vivo.

Changes during and following GnRHa treatment in endometriosis patients have been associated with the cell apoptosis. It have been shown that the apoptosis induced by GnRHa in EEC is mediated by the Bax/Bcl-2 pathway [12]. However, there is little information regarding the involvement of the proteome in this biological process. Herein, compared the levels of protein expression in the eutopic endometrium of endometriosis patients before and after GnRHa treatment using Western blotting and immunochemistry and conducted a comprehensive proteomics analysis between GnRHa treated and control patients to understand the molecular mechanisms underlying the effects of GnRHa on this disease. To our knowledge, this study is the first to focus on the proteomics of the eutopic endometrium in response to GnRHa therapy, showing evidence of GnRHa-related protein changes in the eutopic endometrium at a global level and in the same genotype. Among the differentially expressed proteins identified in the present study, 12 proteins were up-regulated, and 14 proteins were down-regulated. Most of these proteins were described as differentially expressed in previous conventional studies. We will discuss a few of these proteins.

GRP78 is a chief regulator of endoplasmic reticulum (ER) function. GRP78 also acts as an anti-apoptotic factor for protection against ER-stress-induced cell death. Consequently, GRP78 is induced in a wide variety of cancer cells and drug-resistant cancer cells [20]. Recent studies have also indicated that ER stress alters GRP78 expression playing an important role in the development and progression of a variety of tumors. Moreover, the localization of GRP78 to the plasma membrane suggest that this protein might also play a role in endometrial cancer development and progression, indicating a novel target for the treatment of endometrial cancer [21]. Taken together, these studies indicate an important role for GRP78 in the treatment of endometriosis.

## Conclusion

The results of the present study showed that GRP78 expression is significantly decreased in eutopic endometrial epithelial cells after LA treatment, and GRP78 knockdown enhanced LA-induced cell apoptosis, while the overexpression of GRP78 suppresses LA-induced apoptosis in eutopic endometrial epithelial cells. These results suggest that GRP78 knockdown might be a useful in the development of GnRHa drugs that promote endometrial epithelial cell apoptosis.

Furthermore, these findings also suggest that GnRHa might effectively promote the apoptosis of endometrial cells both in vitro and in vivo, and induce endometrial epithelial cell apoptosis through the downregulation of GRP78. This knowledge could contribute to a better understanding of the mechanisms underlying the therapeutic action of GnRHa and the medical treatment of endometriosis.
